# Intellectual capital and financial performance: A comparative study

**DOI:** 10.3389/fpsyg.2022.967820

**Published:** 2022-08-23

**Authors:** Shahid Ali, Ghulam Murtaza, Martina Hedvicakova, Junfeng Jiang, Muhammad Naeem

**Affiliations:** ^1^School of Management Science and Engineering, Nanjing University of Information Science and Technology, Nanjing, China; ^2^Department of Commerce, The Islamia University of Bahawalpur, Bahawalpur, Pakistan; ^3^Department of Economics, Faculty of Informatics and Management, University of Hradec Králové, Hradec Králové, Czech Republic; ^4^Department of Business Administration, Iqra University, Islamabad, Pakistan

**Keywords:** intellectual capital, financial performance, value-added intellectual coefficient, human capital, structural capital, capital employed

## Abstract

Intellectual Capital (IC) is a driving force behind the financial performance of non-financial firms. Investing in intellectual and physical capital allows companies to optimize their financial performance by maximizing resource utilization. This study aims to determine whether IC efficiency impacts the financial performance of listed Pakistani and Indian companies between 2010 and 2020. Return on Assets (ROA) and Return on Equity (ROE) are used to calculate financial performance, and IC is calculated using the modified Value-Added Intellectual Coefficient (MVAIC) model. Regression analysis is performed using the STATA software developed by the South Texas Art Therapy Association. Human Capital (HC), Structural Capital (SC), and Capital Employed (CE) have a significant impact on Pakistani and Indian firms’ financial performance. Resource-based theory (RBT) supports these findings. The findings should provide management with a prompt to improve financial performance and emphasize the importance of IC. A rare study has addressed the impact of IC on firm financial performance using the MVAIC model, rather than the VAIC model, in Pakistan and India.

## Introduction

Studies on non-financial firms in Pakistan and India have revealed a high failure rate due to weak support, inadequate managerial skills, and limited resources (Khan et al., 2019; Al-Qahtani and Elgharbawy, 2020). Further, they lack the technological and financial capability to combat external and internal risks like Pakistan’s political instability, increased taxes, increased import costs, Indian companies’ high debt ratios, India’s high tariffs and protectionist policies, local content regulations in India, state-specific regulations, and competition (Motta and Sharma, 2020). As a result, organizations in Pakistan and India confront various difficulties, requiring both real and intangible resources, skills, and competence to improve their performance (Ali et al., 2020). As a result of the high cost and lack of convenience, emerging economies such as Pakistan and India are seeking intangible skills, notably IC, to enhance their performance.

Globalization has, however, evolved from an industry-based economy to one based on knowledge, and enterprises have focused on developing their intangible assets to compete and produce value for long-term success (Gupta et al., 2020). A knowledge economy is defined by the Organization for Economic Cooperation and Development [OECD, OECD Staff, Development (OECD) Staff, Organization for Economic Cooperation and Development, OECD Group of Financial Statisticians, Development. Committee on International Investment, and Multinational Enterprises, 1996] as: “an economy in which the fundamental drivers of development and expansion are the generation, production, and utilization of knowledge; hence, the name knowledge-based economy originated.” Nassau William Senior first described IC as a concept in 1836 (Senior, 1836). IC plays a significant role in a firm’s wealth creation, but it is not documented on its balance sheet like tangible assets (Xu and Liu, 2020). Intangible assets include employee skills, research, and development, systems, and databases that provide a competitive advantage (Ahmed et al., 2019; Kweh et al., 2019). According to Cuozzo et al. (2017), the IC is a value not only in monetary gain but also includes environmental, social, and economic matters. Shahwan and Habib (2020) said that IC is the sum of all employee competencies and skills that generate wealth for the firm.

A firm’s value was determined primarily by physical assets in the early days of business. Intangible assets were primarily speculative in nature and played a minor role. Contemporarily, tangible assets’ importance has declined while investments in information and other intangibles have increased. Executives in organizations and governments have mainly focused on intangible assets to generate sustainable competitive advantage (Cegarra-Navarro and Sánchez-Polo, 2010). According to Gupta et al. (2020), more than 80% of corporate value is derived through intangible assets, including inventions.

Firms have realized the importance of knowledge and the need to efficiently manage it, contributing to the development of the knowledge economy. The relevance of IC has grown rapidly because of the rising significance of the knowledge economy (Cabrita, 2009). According to Gupta et al. (2020), IC has three components: (i) HC represents employees’ commitments, competencies, motivation, and loyalty, (ii) SC denotes infrastructures, procedures, and configurations, and (iii) RC stands for firms’ relationships. The innovativeness of the business comes from the relationships between workers, groups, and organizations (Sardo et al., 2018). Without high-quality workers, it is almost impossible to achieve success through financial assets, solid infrastructure, and new technologies (Tran et al., 2020). HC plays a significant role in increasing efficiency and firm productivity (Anifowose et al., 2018). It is important for industries where companies compete for innovation and advancement, like banking and pharmaceuticals (Haris et al., 2019). Firms with strong SC provide opportunities for their employees to use their skills and knowledge to gain a competitive advantage (Gupta et al., 2020). On the other hand, companies with poor SC cannot meet their goals (Widener, 2006). RC helps to strengthen the firm’s external links (Oppong et al., 2019). Investments in advertising and selling are key sources for creating RC. Firms with high RC form more relationships with partners, boosting their interdependence. Mostly, interdependence-related social interactions grow trust, which occasionally takes the place of explicit contracts (Oppong et al., 2019).

The importance of IC cannot be overlooked, particularly in the case of Pakistani and Indian non-financial sectors, because employment and efficient utilization of IC is now regarded as the most critical and pivotal factor in the success of the non-financial sector. This is since non-financial sectors equipped with IC tend to provide high-quality services through continuous training, brand development, system upgrades, improved processes, and strengthening of stakeholders’ relationships. Therefore, IC’s effective and efficient management becomes of utmost significance for the non-financial sector to operate competitively. It is evident from an international perspective that the non-financial industry around the globe is taking maximum advantage of IC and technology to maximize their shareholders’ wealth (Asif et al., 2020). This trend now, particularly, is not uncommon in Pakistan and India. Accordingly, the non-financial sector is now prioritizing IC and building relationships with stakeholders over merely employing many employees to achieve higher financial performance. RBT emphasizes the importance of tangible and intangible resources (IC) to achieve organizational objectives.

Given IC’s significance and spinal role in the non-financial sector, this study aims to extend its scope by employing widely used Pulic’s MVAIC technique. Therefore, this study investigates In Considering India and Pakistan as developing economies, no comparative studies have been conducted to determine the impact of IC on a firm’s financial performance from 2010 to 2020.

The contributions of this study are as follows. Firstly, this research examines the influence of IC on non-financial firms’ financial performance. Studies on IC have been conducted primarily in a single country or region, with a limited number of comparative studies (Young et al., 2009). Secondly, this study conducted a comparative analysis in the context of two growing Asian nations. Thirdly, it reveals the IC component which most impacts a firm’s financial performance. Fourthly, most previous studies were conducted in developed rather than developing countries. So, this research is performed in Pakistan and India. Ramírez et al. (2020) investigated Spanish SMEs and discovered that intangible resources positively impact firm financial performance. Bolívar and Chrispeels (2011) pointed out that most studies mainly focused on SC and HC rather than RC. The MVAIC model considers the firm’s RC and innovation capital (Smriti and Das, 2018; Xu and Li, 2019). As a result, to bridge the gap, this research employs MVAIC. Finally, this study assists listed firms’ management in enhancing their financial performance and provides vital insights for policymakers in achieving their objectives.

Following is an overview of the remainder of this paper. An overview of the literature is presented in section two, followed by the development of hypotheses in section three. A summary of the results is presented in section four, and a discussion of these results is presented in section five. Lastly, the conclusion discusses the study’s limitations, contributions, and implications.

## Literature review

### Theoretical foundation

One generally accepted theory is RBT is used in this study. Wernerfelt (1984), who formalized this theory, argued that “to the corporation, resources and products are the two sides of coins.” RBT claims that the firm has the resources to achieve objectives and that it can lead the company to improve long-term performance. Valuable and scarce resources can be directed to achieve goals. According to Barney and Arikan (2001), “resources are the tangible and intangible assets firms use to develop and implement their policies.” This theory is based on resource heterogeneity (diversity) and resource immobility (Nothnagel, 2008). RBT is an appropriate way to describe the study of IC, particularly regarding the link between IC and firm performance. Intangible assets of firms are grouped into three major groups by IC: HC, SC, and RC (Kristandl and Bontis, 2007). According to Pulic and Kolakovic (2003), each organization has specific knowledge, skills, values, and solutions—intangible resources—that may be converted into market “value.” Intangible resource management may help firms achieve objectives, enhance productivity, and increase market value. Pulic and Kolakovic's (2003) argument is consistent with Barney and Arikan (2001) reasoning when discussing the link between the two assumption resources in RBT.

### Measuring IC–MVAIC

Edvinsson and Malone (1997) initially developed IC efficiency components in the Skandia model. Human Capital Efficiency (HCE), Structural Capital Efficiency (SCE), and Capital Employed Efficiency (CEE) were calculated using a model developed by Ante Public (1998, 2000, 2003, and 2005). The value-added intellectual capital (VAIC) measurement method is widely accepted and used to assess Intellectual Capital Performance (ICP). The VAIC model was used to correlate firm performance. Researchers have linked IC to firm performance (Zulkifli et al., 2017). Nevertheless, VAIC has various shortcomings that may significantly impair IC valuation (Bayraktaroglu et al., 2019). Studies have shown that VAIC does not measure RC, a major influencer. In addition to being a pillar of IC, RC may also serve as a mediator between HC and IC. As a result, researchers have proposed using MVAIC to compute SCE and RCE (Tiwari and Vidyarthi, 2018; Saddam and Jaafar, 2021). The study also utilized a modified and improved VAIC to better represent the outcomes of SCE and RCE. VAIC is unable to evaluate firms with a negative book value (BV) or operating income, resulting in a negative value-added (VA). To achieve meaningful analysis, these firms must be excluded from the sample (Chu et al., 2011). To resolve the limitations of the original VAIC model and to quantify value-added more completely, some researchers developed the MVAIC model (Xu and Wang, 2018; Dalwai and Mohammadi, 2020). HC, SC, and RC are included in MVAIC, along with the physical components of IC CE (Xu and Wang, 2018; Dalwai and Mohammadi, 2020).

Several studies failed to show any crucial association between HCE, SCE, RCE, and CEE (Maditinos et al., 2011). Ghosh and Mondal (2009) found no correlation between IC and financial performance. This study aims to broaden the perspective on IC among Pakistani and Indian non-financial firms by exploring the effects of MVAIC and its components on firm financial performance.

***H1***: MVAIC has a significant impact on firm financial performance.

### Human capital

HC is the central and vital element of IC (Duho and Onumah, 2019). This reflects the value of company employees’ knowledge, data, and resources. It also applies to an organization with human potential for addressing business issues and optimizing wealth. It incorporates the non-inherited workers’ skills, information, expertise, and experience (Ahangar, 2011). HC is essential for increasing efficiency and profitability (Hamdan, 2018; Alamanda and Springer, 2019). Based on the preceding discussion, the following hypothesis is formed.

***H2***: HCE has a significant effect on firm financial performance.

### Structural capital

SC is also known as organizational capital (Nawaz, 2017). It is the solid foundation that enables the company to work systematically. Even if employees leave, this capital remains with the firm (Poh et al., 2018). It consists of innovations, frameworks, processes, information, designs, databases, structures, composition, and policies that give an edge to firms (Gupta et al., 2020). All infrastructural assets, Intellectual Property Rights (IPR), databases, R&D activities, software, hardware, corporate culture, functions, and everything else that supports the employees’ productivity are included in SC (Appuhami, 2007). As a result, the current study proposes the following hypothesis to assess the impact of SC on financial performance.

***H3***: SCE has a positive influence on firm financial performance.

### Relational capital

The value a corporation develops through relationships with its suppliers, employees, customers, government, and shareholders is known as RC. It is also called customer capital and keeps society and businesses connected. RC links firms to the outside world and gathers knowledge about their customers’ needs and desires (Grasenick and Low, 2004). Firms can generate customer capital by utilizing employee expertise and knowledge to provide better services (exploitation processes) and/or establish new external communities of practice (exploration process). Moreover, Rochmadhona et al. (2018) emphasized that the ability to innovate and improve customer service is vital to a firm’s success. The following hypothesis based on the preceding discussion is as under.

***H4***: RCE has a positive impact on firm financial performance.

### Capital employed

Capital is required to provide goods and/or services to generate profits. Conversely, CE is the entire amount of money invested in a company’s fixed and current assets (Gupta et al., 2020). Pulic (2000) claimed that efficient utilization of physical capital increases the performance of companies. Developing companies mostly focus on physical capital. Haris et al. (2019) found CEE’s significantly positive effect on firm financial performance. As a result, the following hypothesis is developed.

***H5***: CEE has a positive effect on firm financial performance.

### IC and firm financial performance in Pakistan

A study by Rehman et al. (2012) examined the performance of 20 conventional and Islamic banks based on the effect of three components of IC. They used predictive analysis and concluded that VAIC positively influences banks’ performance. In comparison with the components of IC’s performance, all banks have a higher HCE value. It reveals that HCE contributed to 70 to 80% of value creation. Therefore, investing in HC pays more than investing in other IC components. The study used multiple regression to analyze the association between IC and 78 financial firms’ performance from 2008 to 2013 (Ahmad and Ahmed, 2016); Financial firms, including Pakistan’s banks, insurance companies, and mutual funds firms.

In comparison with other components of IC, HC has the greatest impact on financial companies’ performance. To increase their performance, financial companies invest in HC. There is a significant and positive relationship between HCE and CEE, whereas SCE has no effect on performance, and VAIC has a significant and positive relationship with performance (Ahmad and Ahmed, 2016). An investigation of Moradaba companies’ IC and their performance conducted by Ahmad and Bin Mohammad (2019) used predictive analysis and showed that IC components HCE, SCE, CEE, and VAIC significantly influence their financial performance.

According to Ul Rehman et al. (2013), IC positively impacted 21 insurance companies’ performance between 2009 and 2010. They used a questionnaire survey for data collection. After applying Kaiser–Meyer–Olkin (KMO), they found that HCE is significantly positive, while SCE and CEE insignificantly influence firm financial performance, respectively.

Kharal et al. (2014) focused on the financial performance of 12 Pakistani oil and gas companies to determine the fact of IC from 2005 to 2013. They employed Pooled OLS analysis technique and found that IC significantly positively affects financial performance. Xu and Li (2019) used a sample of 29 Chinese and 20 Pakistani banks from 2010 to 2019 to determine the IC influence on banks’ financial performance using the VAIC method. Findings showed that both countries’ banks’ IC positively influences their financial performance, while CEE has the highest contribution to banks’ financial performance. Chinese bank’s profitability is driven by SCE, while HCE drives the bank profitability in Pakistan. Lagged IC can positively impact the profitability of a bank. In emerging Asian markets, there is a need to increase investment in IC to improve bank performance. Shumaila and Afza (2014) explored the association between firms’ IC and performance from 2009 to 2011. They studied a hundred textile and chemical companies and used correlation, a fixed-effect model, and the Hausman test. They found that IC and its components, HCE, CEE, and SCE, positively affect companies’ financial performance.

### IC and firm financial performance in India

Based on the financial performance of 101 banks from 2009 to 2018, Waqar et al. (2020) examined the effect of IC on the financial performance of India’s financial industry. They used multiple regression and the VAIC method to analyze the data. They concluded that HCE, CEE, and SCE all positively impact banks’ financial performance, with CEE having the greatest impact. In Ovechkin et al. (2021), VAIC significantly positively affected company financial performance. The size of a firm has a significant negative impact on its performance. Profitability is lower for large companies. The researchers investigated the association between IC components and banks’ performance using ordinary least squares, random effects, and fixed effects. The analysis results indicated that HCE, SCE, CEE, and advertising efficiency all positively impact bank performance. According to Smriti and Das (2018), 710 service and manufacturing firms were studied to determine the impact of IC on their performance. A variance factor analysis was conducted in this study, as well as a generalized method of moments, to examine the relationships between variables. According to her research, HCE has a marginally negative impact on ROA and ROE. In contrast, SCE has a negligible influence on firm financial performance, while CEE significantly impacts these firms’ effectively utilize their SC and tangible assets. Due to negative associations, investors are still reluctant to invest in human assets.

Infrastructure and real estate firms have a major focus on CEE to gain a competitive advantage, and these companies’ profitability is high due to CEE. Singla (2020) investigated the association between IC and firm financial performance in infrastructure and real estate sectors from 2008 to 2017. They used variance inflation analysis and panel regression analysis. They found that CEE positively influences financial performance. Pal and Soriya (2012) analyzed the association between ICE and firm financial performance for hundred and five pharmaceutical and hundred and two textile firms from 2009 to 2010. They used VAIC (OLS) regression and found a positive association of IC with profitability measured in terms of ROA.

### Theoretical framework

**Figure 1:**
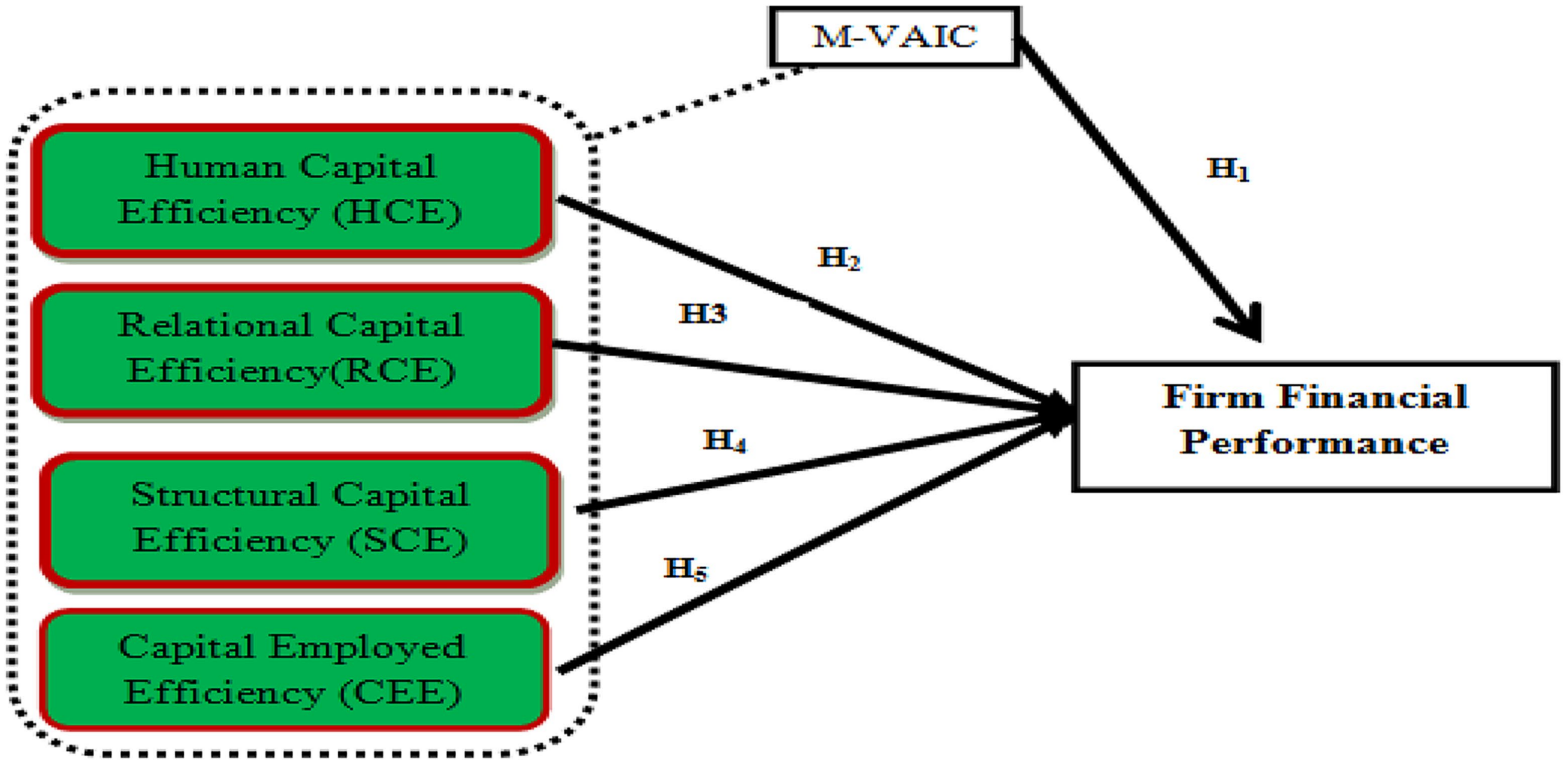
Theoretical framework.

The theoretical framework of the present study is mentioned in Figure 1.

## Research methodology

### Population and sample size

All the listed Pakistani and Indian non-financial firms are the potential population of this study. Five thousand four hundred thirty-nine and 540 companies are listed on the Bombay Stock Exchange (BSX) and Pakistan Stock Exchange (PSX), respectively. Firms that have not remained active from 2010 to 2020 have been removed. In addition, firms with IC, CEE, and financial performance data have been chosen. Then, a sample of 140 firms based on the highest market capitalization from different sectors is selected. The data were obtained from annual reports, stock exchanges, and India Screener.

### Variables and their measurement

To measure IC and its components, most studies have used the VAIC model (Ekwe, 2012; McDowell et al., 2018). The value added of a firm’s financial performance is measured by the contribution of both IC and physical capital. Therefore, this study employs MVAIC rather than the VAIC model. Table 1 contains the detail of variables and their measurements.

**Table 1:** Variables measurement and abbreviations.

Sr.	Variables	Types	Measures	Abbreviation
1	Return on Assets	Dependent Variable	Net Income / Total Assets	ROA
2	Return on Equity	Dependent Variable	Total Income / Shareholder’s Equity	ROE
3	Human Capital Efficiency	Independent Variable	Value Added / Human Capital	HCE
	a) Value Added		Net Sales - Total Expenses	VA
	b) Human Capital		Employee Costs	HC
4	Structural Capital Efficiency	Independent Variable	Structural Capital / Value Added	SCE
	a) Structural Capital		Value Added-Human Capital	SC
5	Relational Capital Efficiency	Independent Variable	Relational Capital / Value Added	RCE
	a) Relational Capital		Marketing + Selling + Promotion + Donations	RC
6	Capital Employed Efficiency	Independent Variable	Value Added/ Capital Employed	CEE
	a) Capital Employed		Total Assets-Intangible Assets	CE
7	Firm Size	Control Variable	Log of Total Asset	Size
8	Firm Leverage	Control Variable	Total Debts / Total Assets	Lev
9	Firm Age	Control Variable	Log of number of years of commencement of business of firm	Age

### Model specification

The association between IC and firm performance has been measured through the following models:


(1) $$\begin{array}{l} ROA_{it}{}_{=}\beta_{o}+\beta_{1}HCE_{it}+\beta_{2}RCE_{it}+\beta_{3}SCE_{it}+\beta_{4}CEE_{it}+\\ \;\;\;\;\;\;\;\;\;\;\;\;\;\;\;\;\;\beta_{5}Size_{it}+\beta_{6}LEV_{it}+\beta_{7}age_{it}+{€}_{it} \end{array}$$

(2) $$\begin{array}{l} ROE_{it}=\beta_{o}+\beta_{1}MVAIC_{it}+\beta_{2}Size_{it}+\\ \;\;\;\;\;\;\;\;\;\;\;\;\;\;\;\;\;\;\;\;\;\beta_{3}LEV_{it}+\beta_{4}age_{it}+{€}_{it} \end{array}$$

(3) $$\begin{array}{l} ROA_{it}{}_{=}\beta_{o}+\beta_{1}MVAIC_{it}+\beta_{2}Size_{it}+\\ \;\;\;\;\;\;\;\;\;\;\;\;\;\;\;\;\;\beta_{3}LEV_{it}+\beta_{4}age_{it}+{€}_{it} \end{array}$$

(4) $$\begin{array}{l} ROE_{it}=\beta_{o}+\beta_{1}HCE_{it}+\beta_{2}RCE_{it}+\beta_{3}SCE_{it}+\beta_{4}CEE_{it}+\\ \;\;\;\;\;\;\;\;\;\;\;\;\;\;\;\;\;\;\;\;\beta_{5}Size_{it}+\beta_{6}LEV_{it}+\beta_{7}age_{it}+{€}_{it} \end{array}$$

where ROA_it_ = Return on Assets of firm i at time t; ROE_it=_ Return on Equity of firm i at time t; HCE_it_ = Human Capital Efficiency of firm i at time t; SCE_it_ = Structural Capital Efficiency of firm i at time t; RCE_it_ = Relational Capital Efficiency of firm i at time t; CEE_it_ = Capital Employed Efficiency of firm i at time t; Size_it_ = Size of firm i at time t; LEV_it_ = Leverage of firm i at time t; Age_it_ = Age of firm i at time t; €_it_ = error term.

## Data analysis

This section divides the data analysis into three subsections. The first section offers a comparison of descriptive statistics. The second and third sections provide correlation and regression analyses of Pakistani and Indian firms, respectively.

### Descriptive statistics

The descriptive statistics for the variables are summarized in Table 2. This table provides information regarding the number of observations, the mean, standard deviation, minimum, and maximum of each variable. Based on the table, Pakistani firms have an average return on assets of 0.09, while Indian firms have an average return on assets of 0.109, which means that the average return on assets of Pakistani firms is 9%, while the average return on assets of Indian firms is approximately 11%. The value of ROA of Pakistani firms is 0.369, while Indian firms are 1.532. Results also show that the mean value of Pakistani firms’ ROE is 0.178 and Indian firms are 0.20, which means that the average return from the equity of Pakistani firms is 17% and Indian firms are 20%. For Pakistani firms, the mean of HCE is 3.639; for Indian firms, its value is 3.781. HCE’s maximum value in Pakistani firms is 14.122, and its significance in Indian firms is 18.859. MVAIC (2.733, 2.79) has reported the biggest standard deviation for the independent variables, indicating that the MVAIC scores of the sample firms vary. The findings demonstrate that HCE has the greatest mean value among ICE components in Pakistan and India, i.e., 2.573 and 18.873, followed by SCE, RCE, and CEE.

**Table 2:** Descriptive statistics.

Variable Name	Obs	Pakistani firms				Indian firms			
		Mean	Std. dev	Min	Max	Mean	Std. dev	Min	Max
ROA	770	0.09	0.088	-0.1	0.369	0.109	0.101	-0.197	1.532
ROE	770	0.178	0.256	-0.503	1.496	0.2	0.21	-0.382	3.006
HCE	770	3.639	2.573	0.481	14.122	3.781	1.862	-0.224	18.859
SCE	770	0.609	0.233	-0.491	0.941	0.673	0.162	-0.369	0.947
RCE	770	0.162	0.179	0	0.833	0.289	0.327	-0.108	2.667
CEE	770	0.493	0.351	0.057	2.382	0.43	0.329	-0.023	4.161
MVAIC	770	4.913	2.733	0.785	15.73	5.173	2.079	0.372	20.978
Age	770	40.561	21.324	7	153	47.649	28.039	5	136
Size	770	7.961	1.376	5.857	10.631	5.015	1.371	1.579	10.756
Lev	770	0.471	0.198	0.051	0.94	0.446	0.196	0.039	0.935

Furthermore, the mean values of HCE, SCE, and RCE in Pakistan and India are higher than the mean values of CEE, which indicates that Pakistani and Indian companies create IC value *via* intangible assets as opposed to physical components, i.e., CEE. The results of this study are consistent with previous studies that indicate that IC, rather than physical capital, provides greater value to firms in developing countries (Pulic, 2004; Zeghal and Maaloul, 2010; Majumder et al., 2021; Murali et al., 2021). The greater mean value of company age in India indicates that Indian enterprises have more experience or average age than Pakistani firms. Pakistani companies have higher leverage mean value than India. It demonstrates that Pakistani companies take more debt than Indian firms.

### Correlation analysis of Pakistani firms

The correlation matrix shown in Table 3 illustrates the relationship between independent variables. Multicollinearity occurs when the correlation coefficient exceeds 0.8 or 0.9, according to Gujarati and Porter (2009). A multicollinearity problem will not be encountered in the regression analysis because none of the independent variables has a high association with one another. In this analysis, the strongest association between CEE and Leverage is 0.222. All other independent variables have a correlation coefficient of less than 0.8.

**Table 3:** Correlation analysis of Pakistani firms.

Variable	HCE	SCE	RCE	CEE	MVAIC	Age	Size	Lev
HCE	1.000							
SCE	0.015	1.000						
RCE	-0.255	-0.253	1.000					
CEE	-0.011	0.019	0.229	1.000				
MVAIC	0.085	0.040	-0.162	0.140	1.000			
Age	-0.135	-0.085	0.204	0.112	-0.107	1.000		
Size	0.029	0.028	-0.197	-0.190	-0.008	-0.216	1.000	
Lev	-0.160	-0.181	0.175	0.222	-0.121	-0.080	0.062	1.000

### Regression analysis of Pakistani firms

IC’s impact on Pakistani enterprise performance was assessed through regression analysis. In the current study, three control factors have been employed to limit the effect of these factors on the financial performance of firms (Size, Leverage, and Age). An evaluation of the MVAIC is conducted separately, and its impact on the firm’s performance is determined. This study examines the effects of HCE, RCE, SCE, and CEE on profitability. The regression findings are summarized in Table 4. It was found that the Predicting variables HCE, SCE, CEE, and MVAIC had a significant impact on ROA and ROE. There is a significant positive correlation between ROA and ROE and HCE, SCE, CEE, and MVAIC. The result is similar to those reported by Ahmad and Ahmed (2016). In contrast, RCE has a negative relationship with ROA and no relationship with ROE. It is consistent with the previous literature (Battisti et al., 2021).

**Table 4:** Regression analysis of Pakistani firms.

Variables	HCE		SCE		RCE		CEE		MVAIC	
	ROA	ROE	ROA	ROE	ROA	ROE	ROA	ROE	ROA	ROE
HCE	0.0140*** (0.001)	0.0339*** (0.003)								
SCE			0.188*** (0.011)	0.549*** (0.035)						
RCE					-0.000796 (0.017)	0.00621 (0.054)				
CEE							0.174*** (0.006)	0.561*** (0.019)		
MVAIC									0.0163*** (0.001)	0.0421*** (0.003)
Constant	0.121*** (0.020)	0.0479 (0.064)	0.0573*** (0.020)	-0.174*** (0.062)	0.195*** (0.021)	0.226*** (0.066)	0.0889*** (0.015)	-0.113** (0.046)	0.0840*** (0.019)	-0.0596 (0.062)
Age	0.000272** (0.000)	0.00068 (0.000)	0.000220* (0.000)	0.000653* (0.000)	0.0000245 (0.000)	0.0000694 (0.000)	-0.000237** (0.000)	-0.000760** (0.000)	0.000280** (0.000)	0.000744* (0.000)
Size	-0.00347* (0.002)	-0.0125* (0.006)	-0.00386** (0.002)	-0.0137** (0.006)	-0.00328 (0.002)	-0.0118* (0.007)	0.00497*** (0.002)	0.0145*** (0.005)	-0.00241 (0.002)	-0.00975 (0.006)
Lev	-0.137*** (0.014)	0.167*** (0.045)	-0.127*** (0.013)	0.213*** (0.041)	-0.169*** (0.015)	0.0900* (0.048)	-0.243*** (0.011)	-0.148*** (0.033)	-0.140*** (0.013)	0.166*** (0.042)
Observations	770	770	770	770	770	770	770	770	770	770
R-squared	0.308	0.12	0.383	0.247	0.149	0.009	0.58	0.543	0.395	0.206

Standard errors in parentheses. ***p<0.01, **p<0.05, *p<0.1.

HCE positively impacts ROA and ROE with R-squared 0.308 and 0.12, respectively, which means that HCE brings a 30% and 12% change in ROA and ROE, respectively. HCE is very important for Pakistani firms to achieve their primary objective in the form of ROA and ROE. It implies that Pakistani firms focus more on employee training, skills, and education. SCE also positively impacts ROA and ROE with R-squared 0.383 and 0.247, respectively. Information, networking expenditures, policies, and frameworks must be managed efficiently to obtain effective benefits. The RCE is negatively associated with ROA but has no impact on ROE. Therefore, companies are advised to focus on maintaining their relationship with stakeholders since stakeholder trust is also an important factor in enhancing the firm’s financial performance.

Furthermore, financial capital should also be given due consideration. CEE also influences firm financial performance significantly because the R-squared of ROA and ROE are 0.58 and 0.543, respectively. Overall, MVAIC significantly impacts firm financial performance ROA and ROE with R-squared 0.395 and 0.206. These assets can improve a company’s financial performance if used properly and consistently. As a result, the conclusion can be formed that overall, IC should be prioritized to acquire a competitive edge and improve business financial performance.

### Correlation analysis of Indian firms

The correlation matrix in Table 5 illustrates the relationship between the independent variables. The correlation analysis will not be affected by multicollinearity since none of the independent variables are highly associated with each other. In this study, MVAIC and RCE have the highest degree of association at 0.231. Besides these two variables, all other independent variables have a correlation coefficient of less than 0.8. Since these variables are related to the predicted variable, they should impact the firm’s financial performance.

**Table 5:** Correlation analysis of Indian firms.

Variables	HCE	SCE	RCE		CEE		MVAIC		Age		Size		Lev		
HCE	1.000														
SCE	0.011	1.000													
RCE	0.070	0.152		1.000											
CEE	0.031	-0.096		-0.008		1.000									
MVAIC	0.074		0.013		0.231		0.177		1.000							
Age	-0.061		0.008		0.176		0.146		-0.003		1.000					
Size	0.135		0.055		0.005		-0.135		0.104		-0.099		1.000		
Lev	-0.056	0.047		0.007		0.022		-0.049		0.015		0.104		1.000	

### Regression analysis of Indian firms

HCE positively impacts ROA and ROE with R-squared 0.255 and 0.138, respectively, which means that HCE brings a 25 and 13% change in ROA and ROE, respectively. HCE has a positive and significant relationship with ROA and ROE, which suggests that HC may contribute to improving company performance. In addition, SCE positively impacts ROA and ROE, with R-squared values of 0.204 and 0.108, respectively. This result is similar to those (Hamdan et al., 2017; Amin et al., 2018; Adegbayibi, 2021). There is a similar result to those found in previous studies (Hamdan et al., 2017; Amin et al., 2018; Adegbayibi, 2021). RCE has no impact on ROA and ROE. This implies that companies should maintain their relationships with stakeholders. Additionally, it is imperative to pay attention to the financial capital simultaneously. Earlier studies have reported similar results (Battisti et al., 2021). Moreover, CEE significantly impacts a firm’s financial performance because the R-squared of ROA and ROE is 0.616 and 0.585, respectively. CEE’s highest R-square means that CEE brings more changes to Indian firm performance. Overall, MVAIC significantly impacts firm performance ROA and ROE with R-squared 0.331 and 0.219. Table 6 summarizes the regression findings.

**Table 6:** Regression analysis of Indian firms.

Variables	HCE		SCE		RCE		CEE		MVAIC	
	ROA	ROE	ROA	ROE	ROA	ROE	ROA	ROE	ROA	ROE
HCE	0.0196***	0.0361***								
	(0.002)	(0.004)								
SCE			0.172***	0.344***						
			(0.020)	(0.044)						
RCE					0.00391	0.0284				
					(0.011)	(0.023)				
CEE							0.219***	0.479***		
							(0.007)	(0.015)		
MVAIC									0.0221***	0.0432***
									(0.001)	(0.003)
Constant	0.121***	0.0523	0.0784***	-0.0435	0.185***	0.167***	0.0745***	-0.0729***	0.0848***	-0.0268
	(0.016)	(0.035)	(0.020)	(0.044)	(0.016)	(0.035)	(0.011)	(0.024)	(0.016)	(0.035)
Age	0.000632***	0.00130***	0.000559***	0.00117***	0.000564***	0.00113***	0.000227***	0.000436**	0.000557***	0.00116***
	(0.000)	(0.000)	(0.000)	(0.000)	(0.000)	(0.000)	(0.000)	(0.000)	(0.000)	(0.000)
Size	-0.0109***	-0.0185***	-0.00831***	-0.0139***	-0.00728***	-0.0120**	-0.000908	0.00212	-0.0110***	-0.0191***
	(0.002)	(0.005)	(0.002)	(0.005)	(0.003)	(0.005)	(0.002)	(0.004)	(0.002)	(0.005)
Lev	-0.139***	0.0948***	-0.158***	0.0592	-0.152***	0.0708*	-0.148***	0.0799***	-0.138***	0.0986***
	(0.016)	(0.036)	(0.017)	(0.037)	(0.018)	(0.038)	(0.012)	(0.025)	(0.015)	(0.035)
Observations	770	770	770	770	770	770	770	770	770	770
R-squared	0.255	0.138	0.204	0.108	0.129	0.039	0.616	0.585	0.331	0.219

Standard errors in parentheses. ***p<0.01, **p<0.05, *p<0.1.

### Hypotheses summary

A summary of accepted and rejected hypotheses is presented in Table 7.

**Table 7:** Hypotheses decision.

Sr. No.	Hypotheses	Decision
H1	MVAIC has a significant impact on firm financial performance.	Accepted
H2	HCE has a significant effect on firm financial performance.	Accepted
H3	SCE has a positive influence on firm financial performance.	Accepted
H4	RCE has a positive impact on firm financial performance.	Rejected
H5	CEE has a positive effect on firm financial performance.	Accepted

## Conclusion, policy implications, and future recommendations

To the researcher’s knowledge, this is the first study to compare IC’s impact on firm financial performance in Pakistan and India. According to this study, IC has a positive effect on the financial performance of firms. The results of this study are consistent with previous literature (Ahmad and Ahmed, 2016; Hamdan et al., 2017; Amin et al., 2018; Adegbayibi, 2021; Battisti et al., 2021). In addition, these results are also supported by the RBT theory, which stresses the importance of intangible resources (IC).

Among the components of the MVAIC, HCE, SCE, and CEE are found to positively influence financial performance, whereas RCE has no significant impact. As companies strive to grow their tangible assets, they must pay equal attention to their intangible assets’ productivity, efficiency, and growth. The importance of IC in the production value of enterprises in a knowledge-based economy cannot be overstated. The IC comprises assets such as human capital, processes, networking and information systems, and stakeholder relationships. These assets are imperative for improving efficiency and reducing costs in a company. The financial performance of companies with strong IC will be boosted due to their ability to manage their assets more effectively and efficiently. A company’s financial success is greatly influenced by IC’s ability to produce value. Additionally, the study indicates that intangible assets can improve the financial performance of firms in Pakistan and India.

IC and performance were positively correlated, justifying corporation investments in HC, SC, RC, and CE. Policymakers must focus on developing skilled HC through training and development. In addition, RC is also significant for firm performance, which indicates that management must focus not only on HC but also on social welfare in the society where the company operates.

Investors can benefit from this study by having social screening intact to maximize their returns. Since RC is a relationship between a corporation and the outside community, a focus on price-taking by corporations cannot sustain itself over a long period. Therefore, empirically, it makes sense for investors to invest in companies that do not just focus on their profits but also serve the society they operate in.

In this study, we focus solely on non-financial sectors in Pakistan and India, but future studies could include financial institutions, leasing, insurance, credit unions, asset management organizations, etc.

## Data availability statement

The original contributions presented in the study are included in the article/supplementary material, further inquiries can be directed to the corresponding author.

## Author contributions

SA: writing—initial draft, writing-analysis, visualization, and validation. JJ and MH: reviewing, editing, and supervision. GM: writing literature review, editing, and methodology. MN: writing literature review and editing. All authors contributed to the article and approved the submitted version.

## Funding

This research was funded by the National Natural Science Foundation of China (71972153). The work was supported by the internal project “SPEV – Economic Impacts under the Industry 4.0 / Society 5.0 Concept”, 2022, University of Hradec Králové, Faculty of Informatics and Management, Czech Republic.

## Conflict of interest

The authors declare that the research was conducted in the absence of any commercial or financial relationships that could be construed as a potential conflict of interest.

## Publisher’s note

All claims expressed in this article are solely those of the authors and do not necessarily represent those of their affiliated organizations, or those of the publisher, the editors and the reviewers. Any product that may be evaluated in this article, or claim that may be made by its manufacturer, is not guaranteed or endorsed by the publisher.
